# Free Schools

**Published:** 1881-01

**Authors:** 


The Bistoury.

ELMIRA, N. Y., JANUARY, 1881.

The BISTOURY is published Quarterly, upon the first of April, July, October and January, at Fifty Cents a year in advance.

### FREE SCHOOLS.

Is the present system of popular education a costly and dismal failure? That is the serious question of the day. We have been so long accustomed to look upon our common school system, with its extravagant, pretentious and often unsanitary buildings; its insufficient corps of underpaid teachers; its intricate system of graded classes, and its appallingly complex and extensive curriculum of studies, as at once the root and flower of our civilization, that it may seem almost sacrilegious to even suspect its efficiency. But the question is pressing for an answer; and many of our best thinkers having mustered courage to grapple with the subject, we may as well prepare ourselves to consider the matter.

On purely abstract grounds, it becomes difficult to defend a free school system at all; for, why should the State provide free education any more than free food or free clothing? Conceding, however, that it is good policy for the State to furnish some education—a matter upon which statesmen seem to be agreed—it is plain that the education so furnished should give to the scholar the ability to read his own language freely and intelligently, to write fluently and legibly, and to use the four fundamental rules of arithmetic with sufficient ease and precision for ordinary business purposes. This might be called a fair education, and one, we believe, that the free school system was originally only intended to embrace, and all that the State should be called upon to supply. Anything beyond this, should be considered as accomplishments, to be paid for by those who earnestly desire them.

Be it understood, that we do not advocate the abandonment, at this point, of further attempts at securing a higher education. By no means. If the mechanic, farmer, or daily laborer can secure a knowledge of philosophy, chemistry, physiology, &c., or can progress so far as what is known as a classical education, so much the better for him and for the community in which he lives. But we would not advise the expenditure of the time, in school, necessary for obtaining such knowledge, if he has determined upon learning some trade in which skill of hands is of more importance than knowledge of the sciences mentioned; because the training of the hands should be early begun, and *any* science can be mastered at home, after working hours, by any student determined to learn. A thorough training in and knowledge of the three branches which we believe it was originally intended that the free school system should embrace, will fit any young man or woman for the ordinary purposes of life, and will enable them to add the accomplishments of learning, through their own efforts, if they so determine.

The *Advertiser* of this city was one of the public journals earliest to advocate this theory of education. Since then, various persons and papers have discussed the matter in the same vein. We are, therefore, not without company when we declare our present system to be open to criticism, and on the following points:

First—It attempts too much. It crowds into the course of study a host of non-essentials, such as Latin, Greek, French, German, Chemistry, Botany, Physiology, Algebra, Astronomy, Drawing and Music, to the detriment of the more important branches of Reading, Writing and Arithmetic.

Second—It fails, almost utterly, in the graded system, which yokes the bright and the dull scholars together, and crushes out all individuality of both teachers and pupils.

Third—It gives the scholars a smattering of a little of everything, and teaches them nothing, thoroughly; so, instead of fitting them for business pursuits, it tends to make them superficial, conceited and discontented.

Fourth—The expense is far too great for the results obtained.

How many of the boys who have graduated from the Grammar Schools and the Free Academy can frame an acceptable business letter, write a decent hand, compute the problems occurring in daily business transactions, and speak the English language? How many? In applying this reasonable test, would it not seem that the time devoted to Chemistry, Physiology, Latin, Greek, and the like, had been better employed in gaining increased knowledge of Reading, Writing and Arithmetic?

We have known Teachers in the Grammar Schools to have had classes in the branches last named, numbering from thirty to forty pupils, who were given one half hour every day to these studies. That represents, say, one minute to every scholar. How much information can be imparted and how much drill can be given in one minute? The intervening time was given to an idiotic system of "diagramming" sentences, called grammar; to singing, drawing, printing, botany, book-keeping, &c. With such a system, the wonder is not so much that the scholars read and speak so badly, but rather, that they do so well.

We have in our city about three thousand* children attending the public schools. Most of them, of course, are of poor parents, and consequently obliged to terminate their education with the Grammar School, and then commence contributing to the support of the family, by their daily labor; consequently, never reach the academic department.

By reason of this, the Academy affords tuition of a higher grade to a select few, who ordinarily are abundantly able to pay for such teaching, and should so do, particularly as it is more a matter of accomplishment rather than a necessity. This academic department, which instructs perhaps 150 pupils, as against the 3,000 that attend the Grammar Schools, costs four times as much, per capita, for tuition as the latter.†

Now, it would seem clear that the parents who can afford to keep their children at school through the academic department, could also afford to pay for the privilege, and so release a large sum of money that might be more judiciously expended in employing more teachers in the lower departments, that more time could be employed in *teaching*, instead of hearing recitations, as now.

That our children do not learn more rapidly is by no means the fault of the teachers. A more honest, faithful and hard-working class of people can no where be found than they. The fault lies with the Boards of Education, that compel them to *try* to do more than it is possible for them to accomplish. No man nor woman can teach 50 pupils‡—by our present methods—do justice to the pupils, and keep themselves out of a hospital or lunatic asylum.

The whole system has gradually grown, from year to year, to such extravagant proportions as to give it the appearance of having been designed for show rather than for genuine educational purposes.

An extensive curriculum of studies, including three or four languages [living and dead] half a dozen "ologies," drawing and music, *looks* well in the published course of study, costs a great deal, but is not worth much in practical use. Our school houses, with their cut stone trimmings, fillagree cornices and turrets, winding stairs, and walnut desks, appear well, and perhaps give the community in which they are located a reputation for great liberality, [likely also the unenviable one for high taxation]; but too often are badly lighted, illy heated, and wretchedly ventilated; are hard to get into and dangerous to emerge from, particularly in times of alarm or excitement from accidental fires. Would not plainer, less elaborate and therefore less costly buildings, constructed for use rather than for show, be better for the minds and bodies of teachers and pupils and of great relief to the pockets of the tax payers?

The graded system, made necessary by the insufficient number of teachers, becomes pernicious because it forces pupils of weak mental capacity beyond their strength, while the vigorous and mentally capable scholar is hindered by his less brilliant companions, so squeezing all into one mold in which all individuality is completely lost. In this manner, the child becomes the part of an immense machine, in which much time is squandered in foolish drills, and where writing books are opened in one time and two motions, while the simplest movements are performed with a mechanical rythm much upon the plan of the military manual of arms. This is all very pretty and perhaps quite enjoyable for the pupil, but when carried to the extent that we have witnessed in some public schools, seems to be not only a loss of time, but vexatious also to teacher andpupil.

The reform that naturally suggests itself is,

1. Cheaper and better constructed school buildings for the purposes intended.

2 The employment of more teachers.

3. A complete revision of the course of study, so that more time is given to reading, writing, and arithmetic, and none to the higher branches designated academic.

4. Abandonment of the academic department.

Sooner or later, this reform must come, else will the people—the taxpayers—refuse to support at great cost, a system of education that does not educate.

---

*In 1874 the average attendance was 2,500.

†By referring to the report of the Board of Education for 1874—the only one in our possession—we find that the cost per pupil, in the Grammar Schools, was $11.34; in the Academy, $46.14.

‡The same report shows that the average number of pupils o each teacher is 53.9.

